# Independent prognostic significance of cell cycle regulator proteins p16^INK4a^ and pRb in advanced-stage ovarian carcinoma including optimally debulked patients: a translational research subprotocol of a randomised study of the Arbeitsgemeinschaft Gynaekologische Onkologie Ovarian Cancer Study Group

**DOI:** 10.1038/sj.bjc.6603531

**Published:** 2007-01-23

**Authors:** S Kommoss, A du Bois, R Ridder, M J Trunk, D Schmidt, J Pfisterer, F Kommoss

**Affiliations:** 1 Dr-Horst-Schmidt-Klinik (HSK) Wiesbaden, Department of Gynecology & Gynecologic Oncology, Ludwig – Erhard – Str. 100, Wiesbaden 65199, Germany; 2 mtm laboratories AG, Heidelberg, Germany; 3 Institute of Pathology, A2/2, Mannheim, Germany; 4 Frauenklinik, Universitätsklinikum Mannheim, Germany

**Keywords:** ovarian carcinoma, immunohistochemistry, p16^INK4a^, pRb, prognosis

## Abstract

The purpose of the study is to test the hypothesis that expression of cell cycle regulatory proteins p16^INK4a^ and pRb is significantly associated with prognosis in ovarian carcinomas. We performed immunohistochemical analysis of p16^INK4a^ and pRb expression and correlated with survival in a series of 300 patients with FIGO stage IIb-IV ovarian carcinoma which were enrolled in a randomized prospective trial evaluating two different platinum and paxlitaxel chemotherapy combinations after radical surgery. p16^INK4a^ negative tumours (17/300; 6%) had a significantly worse prognosis (univariate analysis, *P*<0.001; multivariate analysis: odds ratio 2.41, *P*=0.009). Among p16^INK4a^-positive tumours (283 out of 300; 94%), survival was better for patients with intermediate expression as compared to low or high expression levels (*P*=0.001). High expression levels of pRb were associated with an incremental deterioration of prognosis (univariate analysis, *P*=0.004; multivariate analysis: odds ratio 2.98, *P*=0.002). This observation held also true in the subgroup of optimally debulked patients (*n*=82), in whom the most important established prognostic factor, postoperative residual tumour cannot be applied. In conclusion p16^INK4a^ and pRb are independent prognostic factors in advanced-stage ovarian carcinomas after radical surgery and postoperative chemotherapy. High pRb expression is a significant prognosticator in optimally debulked patients and may hold potential for subgroup stratification in postoperative treatment.

State-of-the-art treatment of advanced-stage ovarian carcinoma includes radical cytoreductive surgery aiming at the removal of all visible tumour, followed by six cycles of platinum/paclitaxel (PT) chemotherapy. The completeness of surgical tumour removal is among the most powerful predictors of patient outcome (du Bois *et al*, 2003). Currently, there are no additional generally accepted prognostic markers independent from the established clinical factors like International Federation of Gynaecology and Obstetrics (FIGO) stage, postoperative residual tumour, patient age and clinical performance status (Crawford *et al*, 2005).

The retinoblastoma (RB) regulatory pathway of cell cycle control is deregulated in virtually all human tumour types (Cobrinik, 2005; Korenjak and Brehm, 2005; Macaluso *et al*, 2005). In a physiological cell state, the ability of the retinoblastoma protein (pRb) to control cell cycle progression via binding members of the E2F transcription factor family is controlled by cyclin D/cdk4-mediated phosporylation. The p16^INK4a^ protein has been shown to inhibit the phosphorylating activity of this functional cyclin D/cdk4complex, thus abrogating the E2F-induced activation of genes important for driving the cell cycle into S-phase progression. Various specific genetic alterations of individual members of the pRB-regulated pathway, including cyclin D, cdk4 and cyclin-dependent kinase inhibitor p16^INK4a^, contribute to the impairment of cell cycle control at the G1 S-phase transition in different tumour types. Deregulated expression of cell cycle promoting cyclins, often resulting from specific tumour-associated gene amplification, represents a common mechanism leading to loss of cell cycle control, as well as genetic loss or transcriptional downregulation of cellcycle inhibitory factors, for example pRb and the CDK inhibitor p16^INK4a^ (Pei and Xiong, 2005; Sharpless, 2005). However, p16^INK4a^ expression has also been found to be strongly upregulated in certain neoplasms, such as cervical cancers and cervical high-grade dysplasias (Klaes *et al*, 2001). In proliferating cells from these cervical precancerous and cancerous lesions, the E7 protein from high-risk human papillomaviruses (HPV) inactivates the pRb–E2F transcription factor complex, thus releasing the negative transcriptional control of the p16^INK4a^ gene mediated by functional pRb–E2F complex. Besides its potential utility as a diagnostic marker for the identification of cervical lesions, overexpression of p16^INK4a^ has been reported to represent a characteristic of mammary carcinomas associated with a poor prognosis (Milde-Langosch *et al*, 2001).

In ovarian cancer, expression of p16^INK4a^ and pRb has been investigated in various studies using immunohistochemistry (Dong *et al*, 1997a, 1997b; Niemann *et al*, 1998; Kusume *et al*, 1999; Sui *et al*, 2000; Milde-Langosch *et al*, 2003; Raspollini *et al*, 2004; Hashiguchi *et al*, 2004). However, the results from these studies provided conflicting information concerning the expression of pRb and p16^INK4a^, and the prognostic significance of differential expression levels of these cellcycle regulatory proteins in ovarian carcinomas. Most of the previous studies are characterised by either a relatively low number of cases analysed, or by a broad heterogeneity with respect to stage and postoperative therapy, or by only limited follow-up data being available. Therefore, this study aimed to test the hypothesis that expression of cell-cycle regulatory proteins p16^INK4a^ and pRb is significantly associated with prognosis in a large, homogeneous series of advanced-stage ovarian carcinomas receiving standardised chemotherapy after radical surgery (du Bois *et al*, 2003).

## MATERIALS AND METHODS

### Study material

After completion of a prospective randomised, multi-centre, phase III study of a total of 798 patients with ovarian cancer, FIGO-stages IIB–IV, comparing cisplatin 75 mg m^−2^ plus 185 mg m^−2^ PT with PT 185 mg m^−2^ plus carboplatin (TC) (du Bois *et al*, 2003), tissue blocks were requested from participating centres for translational research. Between one and 53 paraffin blocks per case were received from a total of 334 study patients. An explorative comparison of patient characteristics between patients with or without available paraffin blocks was performed. Paraffin sections were cut from all blocks and stained with Haematoxylin and Eosin. After review of all slides and exclusion of material which contained no tumour tissues, or which was insufficient for adequate histopathological analysis (32 cases), between one and nine stained sections each from a total of 300 cases of primary invasive epithelial ovarian carcinomas were available for further studies. All slides were reviewed by two experienced gynaecopathologists (FK, DS) who were blinded for the outside diagnoses. All ovarian carcinomas were typed according to the current WHO criteria and classified as either serous, mucinous, endometrioid, clear cell, transitional cell or undifferentiated carcinoma (Scully, 1999; Tavassoli and Devilee, 2003).

### Immunohistochemistry

Sections 2–4 *μ*m were cut from paraffin blocks, deparaffinised in xylene and rehydrated. Antigen retrieval for p16^INK4a^ immunostaining was performed by incubating slides in 0.01 M citrate buffer (pH 6.0) for 40 min at 95°C in a water bath (Pasha, Montone and Tomaszewski, 1995), sections for pRb immunostaining in 0.1 M sodium citrate buffer (pH 6.0) for 20 min in a microwave oven (Shi, Key and Kalra, 1991). For p16^INK4a^ immunostaining, mouse monoclonal antibody clone E6H4 (mtm laboratories, Heidelberg, Germany) was used. A polymer-horseradish peroxidase-based secondary reagent system (Envision+, Dako A/S, Glostrup, Denmark) was used for signal generation following the instructions of the manufacturer. For pRb-immunostaining, mouse monoclonal antibody clone 3C8 (QED Bioscience Inc., San Diego, CA, USA) (Wen *et al*, 1994) was used at a 1:450 dilution. Signal generation was carried out following the VECTASTAIN® ELITE ABC KIT protocol (Vector Laboratories, Burlingame, CA, USA). 3,3′-Diaminobenzidine hydrochloride was used as chromogen for both immunostaining protocols, and slides were finally counterstained with Mayer's Hemalaun. Appropriate positive controls (for p16^INK4a^: cervical high-grade lesions (CIN III), for pRb: breast cancer specimens) and negative reagent controls were included in each staining run. Proliferation activity was measured immunohistochemically using monoclonal Ki-S5 antibody (kindly provided by R Parwaresch, Institute of Pathology, University of Kiel, Germany) as described (Kreipe *et al*, 1993).

### Evaluation of immunostains

p16^INK4a^ and pRb immunostaining intensities (ISI: no staining=0; weak staining=1; medium staining=2; strong staining=3) and percentages of positively stained cells (PPC: 0%=0; <10%=1; 10–50%=2; 51–80%=3; >80%=4) were assessed for each tumour. Negative and weak ISI were considered low pRb, mean and strong ISI were considered high pRb. Proliferative activity as measured by Ki-S5 immunostaining was calculated as percentage of positive cells. Any unequivocal nuclear staining of neoplastic epithelial tumour cells was considered specific.

For p16^INK4a^ evaluation, staining patterns of nuclear only, cytoplasmatic only and combined nuclear/cytoplasmatic were accepted as specific; for pRb any unequivocal nuclear staining of epithelial tumour cells was considered specific. For evaluation of Ki-S5 staining, the respective area showing the highest level of proliferation for each tumour specimen was identified at low microscopical power. Five-hundred tumour cells per slide were then counted at a × 400 magnification.

### Statistical analysis

The exploratory analysis of possible selection bias of the study population was performed by the χ^2^-test (Armitage and Berry, 1994). Survival analysis was performed according to the method of Kaplan and Meier (Kaplan and Meier, 1958), survival times were compared using the log rank test (Mantel, 1966) applying SPSS13 software (SPSS for Windows, Rel. 13.0.1., 2004). Multivariate analyses were performed using the Cox regression (Cox, 1972).

## RESULTS

From a randomised prospective trial of patients receiving standardised postoperative chemotherapy after radical surgery, 300 cases of primary invasive epithelial ovarian carcinoma were selected without following any rule, other than being dependent on availability of material and willingness to cooperate. No significant selection bias was found after explorative analysis of patient characteristics (patient age, Eastern Cooperative Oncology Group (ECOG) clinical performance status, FIGO stage, preoperative extraovarian tumour, postoperative residual tumour) comparing patients with and without available paraffin blocks by χ^2^-analysis (data not shown). The distribution of histological types of ovarian carcinomas according to current WHO criteria after central review of H&E-stained re-cuts from all available paraffin blocks is shown in Table 1.

**Figure 1:**
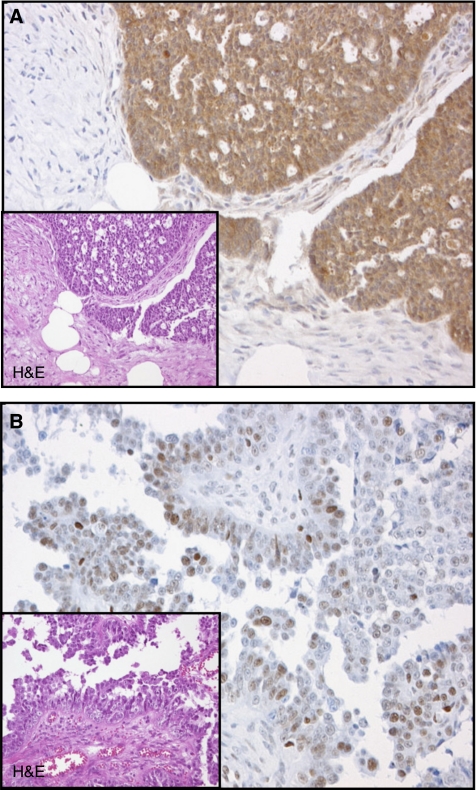
Examples of immunohistochemistry, insets show haematoxylin and eosin stains. (**A**) p16^INK4a^-positive ovarian carcinoma (PPC>80%, ISI 3). (**B**) pRb-positive ovarian carcinoma (PPC 51–80%, ISI 2).

**Figure 2:**
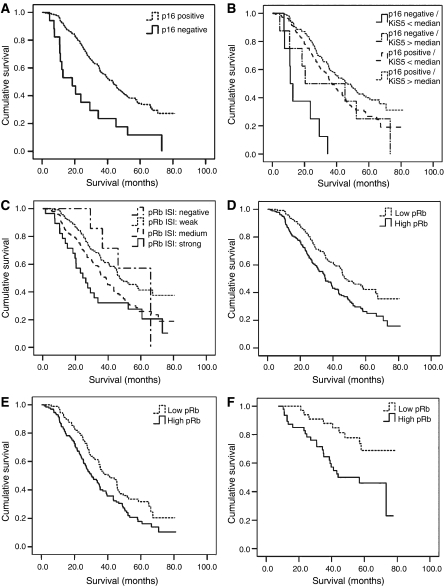
(**A**) Kaplan–Meier survival estimates showed a worse prognosis for p16^INK4a^-negative patients (*n*=17/300; *P*<0.001). (**B**) Among both p16^INK4a^-negative and p16^INK4a^-positive carcinomas, low Ki-S5 proliferation activity was associated with a worse prognosis as compared to high proliferation activity. (**C**) Incremental deterioration of prognosis with increasing pRb ISI (*P*=0.004). (**D**) Patients with high pRb expression (=medium and high ISI, *n*=176) had a worse prognosis as compared to patients with low pRb expression (=negative and low ISI, *n*=124) *P*=0.001. (**E**) Survival curve for the nonoptimally debulked group (*n*=218; *P*=0.015). (**F**) In the subgroup of optimally debulked patients, high pRb indicated a significantly worse prognosis (*n*=82; *P*=0.009).

**Figure 3:**
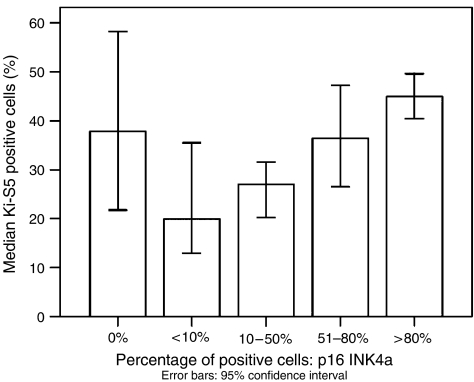
Correlation between p16^INK4a^ expression and proliferative activity as measured by Ki-S5 immunohistochemistry.

Both p16^INK4a^ and pRb immunostains of sufficient quality were available for further analysis from a total of 300 cases. Most cases were p16^INK4a^-positive (283 out of 300; 94%), likewise a majority of cases showed some pRb immunoreactivity (292 out of 300; 97%), ISI and PPC for p16^INK4a^ and pRb, respectively, are given in Table 2. Examples for staining results are given in Figure 1. ISI and PPC were closely covariant (*P*<0.0001) for both markers. p16^INK4a^ and pRb expression levels were found to be independent from the established prognostic factors FIGO stage, postoperative residual tumour and age (Tables 3a and 3b). A statistically significant, however weak, negative correlation between p16^INK4a^ and pRb PPC was observed (correlation coefficient PPC: −0.114, *P*=0.01; ISI: −0.091, *P*=0.08). Kaplan–Meier survival estimates showed a significantly worse prognosis for p16^INK4a^-negative patients (*P*<0.001; Table 2, Figure 2A). p16^INK4a^-negative tumours were of no particular histological type. p16^INK4a^ staining results were independent from established prognostic markers (Table 3a). Among p16^INK4a^-positive tumours (283 out of 300; 94%), survival was better for patients with intermediate expression as compared to low or high expression levels (*P*=0.001; Table 2). Furthermore, p16^INK4a^ expression levels were associated with Ki-S5 proliferation activity. Proliferation activity was higher in ovarian carcinomas with either negative or strong p16^INK4a^ expression, compared to cases with only weak or moderate expression levels (Figure 3). Within the subgroups of p16^INK4a^-negative and p16^INK4a^-positive carcinomas, patients with low proliferation activity as measured by Ki-S5 immunostaining had a lower median survival as compared to those with a high proliferation (p16^INK4a^negative, Ki-S5 low: 11.1 months, Ki-S5 high: 20.4 months; p16^INK4a^positive, Ki-S5 low: 37.2 months, Ki-S5 high: 46.9 months) activity (Figure 2B). Immunohistochemical pRb expression analysis revealed an incremental deterioration of prognosis with increasing pRb ISI (*P*=0.004, Table 2, Figure 2C and D), as well as pRb PPC (*P*=0.001, Table 2). pRb protein expression levels were not associated with Ki-S5 proliferation activity (data not shown). The results of further univariate analyses of established clinical prognosticators are shown in Table 2. Multivariate analysis including FIGO stage, postoperative residual tumour, ECOG performance status, age and Ki-S5 proliferation activity, established an independent prognostic significance of both pRb ISI (odds ratio: 2.98, *P*=0.002) and p16^INK4a^ expression (odds ratio 2.41, *P*=0.009 (Table 4).

For the subgroup of nonoptimally debulked patients (*P*=0,015, Figure 2E), as well as for the subgroup of optimally debulked patients (*P*=0.009, Table 2, Figure 2F), pRb ISI was still found to be a significant prognosticator. Of note, no prognostic information could be derived from the established factors in the latter subgroup. In contrast to pRb ISI, pRb PPC (*P*=0.21) as well as both p16^INK4a^ ISI (*P*=0.49) and PPC (*P*=0.25) were not associated with prognosis in this group of optimally debulked ovarian cancer patients.

## DISCUSSION

In this retrospective study, the prognostic significance of established prognostic factors in ovarian carcinoma was confirmed in a large, uniform treated group of patients with advanced-stage ovarian carcinoma. In addition, an independent prognostic significance of p16^INK4a^ and pRb expression was observed.

Patients with p16^INK4a^-negative tumours had a significantly worse prognosis as compared to patients with p16^INK4a^-positive carcinomas. Interestingly, the evaluation of p16^INK4a^ expression levels as measured by both ISI and PPC provided further prognostic information among the group of p16^INK4a^-positive patients. While survival was optimal among patients showing intermediate p16^INK4a^ expression levels in the tumour tissue, a worse prognosis was observed both in patients with low, as well as with high p16^INK4a^ expression levels. These observations might indicate that loss of cell cycle control characterised by a complete lack of p16^INK4a^ expression defines a relatively small, but distinct subgroup of advanced-stage ovarian cancer patients with an unfavourable prognosis. Furthermore, similar to the situation in cervical cancer, strong upregulation of p16^INK4a^ protein expression obviously reflects a subset of tumours in which the p16^INK4a^-mediated control of cell cycle progression by regulation of the phosphorylation status of pRb is bypassed by genetic alterations of other essential components of the cell cycle control machinery. Whereas in cervical cancer strong p16^INK4a^ overexpression can be observed in virtually all high-grade dysplastic and cancerous cases as a consequence of functional inactivation of pRb by the E7 protein of high-risk HPV, high p16^INK4a^ protein levels characterise only a subgroup of patients with advanced-stage ovarian cancers associated with a worse prognosis.

In this series, proliferative activity as defined by Ki-S5 immunohistochemistry (Kreipe *et al*, 1993) has been show to be of independent prognostic significance (Kommoss *et al*, 2006). Interestingly, high proliferative activity was observed in tumours completely lacking p16^INK4a^ expression as well as in those showing strong p16 immunoreactivity (Figure 3). In the subgroups of patients with p16^INK4a^-negative and p16^INK4a^-positive cancers, the numbers of high- and low-proliferative tumours as assessed by Ki-S5 immunostaining (Kreipe *et al*, 1993) were evenly distributed (p16^INK4a^ -negative, Ki-S5 low: *n*=8, Ki-S5 high: *n*=8; p16^INK4a^ -positive, Ki-S5 low: *n*=139; Ki-S5 high: *n*=140), thus further validating the finding that lack of p16^INK4a^ expression represents an independent strong negative prognostic factor in patients with advanced-stage ovarian cancers, independent from other key characteristics of cancer cells, as for example, proliferative activity (Tables 3a and 3b). In both latter subgroups, patients with low proliferation had a lower median survival as compared to those with high proliferation, and a p16^INK4a^-negative/low proliferation phenotype was associated with the worst prognosis (Figure 2B).

Conflicting results concerning the prognostic impact of p16^INK4a^ expression in ovarian carcinoma have been reported in the past. While no prognostic significance of p16^INK4a^ immunoreactivity was described by Milde-Langosch *et al* (2003), Sui *et al* (2000), as well as by Kusume *et al* (1999), other reports suggested that high levels of p16^INK4a^ expression predict poor prognosis (Dong *et al*, 1997a). These contrary findings may be due to inherent limitations of some of the previously published studies. While the present collective contains only advanced-stage patients receiving standardized chemotherapy (du Bois *et al*, 2003), series comprising both early and advanced FIGO stages were analysed by others (Dong *et al*, 1997a, b; Niemann *et al*, 1998; Kusume *et al*, 1999; Sui *et al*, 2000; Milde-Langosch *et al*, 2003; Hashiguchi *et al*, 2004). Postoperative therapy was either variable (Dong *et al*, 1997b; Kusume *et al*, 1999; Milde-Langosch *et al*, 2003; Hashiguchi *et al*, 2004) or not mentioned (Dong *et al*, 1997a; Sui *et al*, 2000). Furthermore, case numbers were limited in many of the previous reports (some authors reporting <50 cases (Raspollini *et al*, 2004; Niemann *et al*, 1998). Finally, the comparison of survival data is complicated by the use of various cutoff values for the assessment of p16^INK4a^ expression levels in previous publications.

The assessment of ISI and PPC as used in this study has also been applied in some previous studies (Lamperska *et al*, 2002; Milde-Langosch *et al*, 2003). However, there is still substantial heterogeneity when it comes to a definition of specific cutoff values that are used for the respective scoring of immunoreactivities, thus limiting the comparability of results.

The inverse correlation of p16^INK4a^ and pRb expression levels in our study is in line with the physiological regulation of transcription of these two tumour suppressor genes. Transcription from the p16^INK4a^ promoter is suppressed by functional pRb (Li *et al*, 1994), whereas expression of functional p16^INK4a^ induces downregulation of pRb transcription (Fang *et al*, 1998).

In our study, high pRb expression levels were shown to be significantly associated with a worse prognosis in the homogenous group of patients with advanced-stage ovarian cancers. Our findings confirm the results of a previous study suggesting a potential association of high pRb expression with decreased survival (Milde-Langosch *et al*, 2003). In addition, our analysis suggested that low pRb expression levels may still be associated with a significantly superior survival in the subgroup of optimally debulked patients: five-year survival rates for patients with low pRb expression were 68.8%, compared to 46.1% survival rates in patients characterised by high pRb expression levels.

Of note, Dong *et al*. (1997b) found the prognosis to be worse in FIGO stage I tumours with low pRb expression. In that study, high expression levels of pRb were reported in advanced-stage carcinomas; however, no prognostic significance of pRb expression was reached for that subgroup. These findings further support the relevance of using uniform groups of patients characterised by defined sets of tumours, as well as consistent treatment regimens and follow-up schedules when analysing the potential value and clinical relevance of prognosticators of postoperative disease.

This study is limited by case numbers, especially in clinically important subgroups. Although thorough statistical analysis of the study population was performed, a selection bias cannot be completely ruled out.

Further evaluation using prospective study protocols will have to be performed to validate the results obtained in this retrospectively performed analysis. If confirmed, our findings may be of interest for clinical practice. Optimally debulked patients have a much better prognosis as compared to patients with residual tumour (du Bois *et al*, 2003). However, a significant proportion of patients will relapse and finally die even in this favorable subgroup. Recently, complex gene expression profiles have been described as providing independent prognostic information in advanced stage ovarian cancer. Patients showing an unfavourable gene signature had a significantly worse prognosis. Although a similar trend was observed among optimally debulked patients, the prognostic impact did not reach statistical significance (Spentzos *et al*, 2004, 2005). Thus far it has not been possible to further subdivide completely debulked patients into prognostic subgroups. However, tools to identify different prognostic subgroups in this cohort might offer the opportunity for further clinical studies of aggressive maintenance therapy. The results of our study suggest that if the current findings are confirmed prospectively, the assessment of p16^Ink4a^ and pRb expression levels may prove to be a useful prognostic factor in advanced-stage ovarian carcinomas after standardised chemotherapy including completely debulked patients.

## Figures and Tables

**Table 1:** Histological types of ovarian carcinomas

**Histological type**	***n* (%)**
Serous	135 (45.0)
Mucinous	24 (8.0)
Endometrioid	57 (19.0)
Clear cell	37 (12.3)
Transitional cell	16 (5.3)
Undifferentiated	31 (10.3)
Total	300 (100.0)

**Table 2:** p16^INK4a^ and pRB immunostaining results and clinical prognostic factors vs survival of patients with ovarian carcinoma (univariate analysis)

**Parameter**	**Category**	**Number**		**Five-year survival (%)**	**Median survival months (95%CI)**	***P***
*All patients*						
p16^INK4a^ percentage of positive cells	0%	17	300	11.8	18.5 (7.2–29.7)	0.001
	<10%	33		26.4	33.7 (29.0–33.5)	
	10–50%	69		40.7	45.8 (38.8–53.3)	
	51–80%	47		32.1	45.5 (37.4–53.6)	
	>80%	134		31.2	39.0 (30.6–47.5)	
p16^INK4a^ staining intensity	Negative	17	300	11.8	18.5 (7.2–29.7)	<0.001
	Weak	31		39.6	35.5 (18.9–52.0)	
	Medium	134		37.3	44.6 (37.3–51.8)	
	Strong	118		26.0	35.4 (28.0–42.7)	
p16^INK4a^ positive vs negative	Negative	17	300	11.8	18.5 (7.2–29.7)	<0.001
	Positive	283		32.9	41.2 (35.5–46.8)	
pRb percentage of positive cells	0%	8	300	57.1	66.0 (n.a.)	0.001
	<10%	50		47.3	57.0 (39.4–74.6)	
	10–50%	97		37.2	45.2 (32.3–58.1)	
	51–80%	117		21.4	34.9 (26.1–41.9)	
	>80%	28		15.3	27.1 (16.1–38.1)	
pRb staining intensity	Negative	8	300	57.1	66.0 (n.a.)	0.004
	Weak	116		40.8	46.8 (39.9–53.7)	
	Medium	148		24.8	35.5 (31.5–39.4)	
	Strong	28		20.7	24.4 (15.3–33.6)	
pRb low vs high	Low	124	300	41.7	47.2 (37.8–56.6)	0.001
	High	176		24.0	34.9 (29.4–40.5)	
pRb positive vs negative	Negative	8	300	57.1	66.0 (n.a.)	0.320
	Positive	292		31.6	39.3 (34.3–44.4)	
FIGO stage	IIb-IIIa	35	300	64.5	a	0.001
	IIIb/IV	265		27.8	37.0 (32.1–41.9)	
Postoperative residual tumour	0	82	300	55.8	73.2 (43.6–102.8)	<0.001
	0–10 mm	94		25.4	35.5 (26.6–44.3)	
	11+mm	124		21.0	32.3 (34.3–44.9)	
Preoperative tumour size	<10 mm	48	292^b^	42.3	57.0 (45.3–68.8)	0.017
	>10 mm	244		29.1	35.6 (31.7–39.6)	
Age	<65 yrs	217	300	32.8	43.5 (38.8–48.1)	0.030
	>65 yrs	83		28.3	30.2 (22.4–38.0)	
ECOG	0	133	300	38.8	46.7 (34.7–58.7)	0.004
	>0	137		26.3	34.9 (29.6–40.4)	
Ki-S5	<Median	147	295^c^	25.3	35.3 (30.2–40.6)	0.011
	>Median	148		39.0	45.6 (36.0–55.3)	
						
*Completely debulked patients*						
p16^INK4a^ positive vs negative	Negative	4	82	50.0	34.3 (0.0–73.9)	0.227
	Positive	78		56.1	a	
pRb low vs high	Low	34	82	68.8	a	0.009
	High	48		46.1	44.0 (29.3–58.6)	
Preoperative tumour size	< 10 mm	28	80^b^	59.6	a	0.338
	>10 mm	52		51.2	73.2 (36.0–110.4)	
FIGO stage	IIb-IIIa	26	82	70.0	a	0.195
	IIIb/IV	56		50.3	73.2 (52.4–94.0)	
Age (years)	< 65years	61	82	55.6	73.2 (42.3–104.1)	0.319
	>65 years	21		52.9	a	
ECOG	0	46	82	57.5	73.2 (n.a.)	0.534
	>0	36		55.1	a	

CI=confidence interval; ECOG=Eastern Cooperative Oncology Group; FIGO=International Federation of Gynaecology and Obstetrics; pRb=retinoblastoma protein.

a Median survival could not be calculated, since <50% of patients were dead of disease at the time of analysis.

b Missing preoperative tumour size data for eight patients.

c Exclusion of five cases from further analysis for technical reasons.

**Table 3a:** Patient characteristics (all patients): p16^INK4a^ positive vs p16^INK4a^ negative

		**p16^INK4a^ Negative**		**p16^INK4a^ Positive**		**Total**		
**Parameters**	**Category**	***n***	**%**	***n***	**%**	***n***	**%**	***χ*^2^-test**
Tumour postop	0 mm	4	23.5	78	27.6	82	27.3	*P*=0.911
	1–10 mm	6	35.3	88	31.1	94	31.3	
	>10 mm	7	41.2	117	41.3	124	41.3	
								
Tumour preop^a^	<1 cm	4	26.7	44	15.9	48	16.4	*P*=0.272
	>1 cm	11	73.3	233	84.1	244	83.6	
								
Age	< 65 years	9	52.9	208	73.5	217	72.3	*P*=0.066
	>65 years	8	47.1	75	26.5	83	27.7	
								
FIGO stage	IIb–IIIa	3	17.6	32	11.3	35	11.7	*P*=0.429
	IIIb–IV	14	82.4	251	88.7	265	88.3	
								
ECOG	0	5	29.4	128	45.2	133	44.3	*P*=0.202
	>0	12	70.6	155	54.8	167	55.7	
								


ECOG=Eastern Cooperative Oncology Group; FIGO=International Federation of Gynaecology and Obstetrics; pRb=retinoblastoma protein.

a Missing preoperative tumour size data for eight patients.

**Table 3b:** Patient characteristics (optimally debulked patients): low pRb vs high pRb

		**Low pRb**		**High pRb**		**Total**		
**Parameters**	**Category**	***n***	**%**	***n***	**%**	***n***	**%**	***χ*^2^-test**
Tumour preop^a^	<1 cm	12	36.4	16	34.0	28	35.0	*P*=0.830
	>1 cm	21	63.6	31	66.0	52	65.0	
								
Age	<65 years	24	70.6	37	77.1	61	74.4	*P*=0.507
	>65 years	10	29.4	11	22.9	21	25.6	
								
FIGO stage	IIb–IIIa	9	26.5	17	35.4	26	31.7	*P*=0.391
	IIIb–IV	25	73.5	31	64.6	56	68.3	
								
ECOG	0	20	58.8	26	54.2	46	56.1	*P*=0.675
	>0	14	41.2	22	45.8	36	43.9	

ECOG=Eastern Cooperative Oncology Group; FIGO=International Federation of Gynaecology and Obstetrics; pRb=retinoblastoma protein.

a Missing preoperative tumour size data for two patients.

**Table 4:** Survival of patients with ovarian carcinoma, multivariate analysis

**Parameter**	**Category**	**Number**		**OR (95% CI)**	***P***
FIGO stage	IIb–IIIa	35	300	1	0.003
	IIIb/IV	265		2.45 (CI 1.28–4.69)	
Postoperative residual tumour	0	82	300	1	<0.001
	0–10 mm	94		2.04 (1.33–3.14)	
	>10 mm	124		2.27 (1.50–3.44)	
p16^INK4a^ staining	Negative	17	300	2.41 (1.30–4.46)	0.009
	Positive	283		1	
pRb intensity	Negative	8	300	1	0.002
	Weak	116		1.39 (0.50–3.86)	
	Medium	148		2.42 (0.88–6.70)	
	Strong	28		2.98 (0.96–9.24)	
Age (years)	<65	217	300	1	0.018
	>65	83		1.50 (1.08–2.07)	
Ki-S5	<Median	147	295^a^	1.62 (1.22–2.18)	0.001
	>Median	148		1	

CI=confidence interval; FIGO=International Federation of Gynaecology and Obstetrics; OR=odds ratio; pRb=retinoblastoma protein.

a Exclusion of five cases from further analysis for technical reasons.
